# Effects of Post-UV/Ozone Treatment on Electrical Characteristics of Solution-Processed Copper Oxide Thin-Film Transistors

**DOI:** 10.3390/nano13050854

**Published:** 2023-02-24

**Authors:** Hyeonju Lee, Dongwook Kim, Hyunji Shin, Jin-Hyuk Bae, Jaehoon Park

**Affiliations:** 1 Department of Electronic Engineering, Hallym University, Chuncheon 24252, Republic of Korea; 2 Department of Electrical and Computer Engineering, Inha University, Incheon 22212, Republic of Korea; 3 School of Electronics Engineering, Kyungpook National University, Daegu 41566, Republic of Korea; 4 School of Electronic and Electrical Engineering, Kyungpook National University, Daegu 41566, Republic of Korea

**Keywords:** solution-processed CuO semiconductor, thin-film transistor, post UV/ozone treatment

## Abstract

To realize oxide semiconductor-based complementary circuits and better transparent display applications, the electrical properties of *p*-type oxide semiconductors and the performance improvement of *p*-type oxide thin-film transistors (TFTs) are required. In this study, we report the effects of post-UV/ozone (O_3_) treatment on the structural and electrical characteristics of copper oxide (CuO) semiconductor films and the TFT performance. The CuO semiconductor films were fabricated using copper (II) acetate hydrate as a precursor material to solution processing and the UV/O_3_ treatment was performed as a post-treatment after the CuO film was fabricated. During the post-UV/O_3_ treatment for up to 13 min, the solution-processed CuO films exhibited no meaningful change in the surface morphology. On the other hand, analysis of the Raman and X-ray photoemission spectra of solution-processed CuO films revealed that the post-UV/O_3_ treatment induced compressive stress in the film and increased the composition concentration of Cu–O lattice bonding. In the post-UV/O_3_-treated CuO semiconductor layer, the Hall mobility increased significantly to approximately 280 cm^2^ V^−1^ s^−1^, and the conductivity increased to approximately 4.57 × 10^−2^ Ω^−1^ cm^−1^. Post-UV/O_3_-treated CuO TFTs also showed improved electrical properties compared to those of untreated CuO TFTs. The field-effect mobility of the post-UV/O_3_-treated CuO TFT increased to approximately 6.61 × 10^−3^ cm^−2^ V^−1^ s^−1^, and the on-off current ratio increased to approximately 3.51 × 10^3^. These improvements in the electrical characteristics of CuO films and CuO TFTs can be understood through the suppression of weak bonding and structural defects between Cu and O bonds after post-UV/O_3_ treatment. The result demonstrates that the post-UV/O_3_ treatment can be a viable method to improve the performance of *p*-type oxide TFTs.

## 1. Introduction

Due to their many benefits, including their outstanding electrical characteristics, fast charge mobility, and great optical transparency in the visible light area, oxide semiconductors have received a lot of research attention [1,2,3,4]. Among them, oxide-semiconductor-based TFTs have advantages such as high uniformity over a wide area and versatility of processing and are used in various electronic fields, such as chemical sensors, displays, and memory devices. In addition, oxide semiconductors have received a lot of attention and have been studied as active materials in TFTs owing to their various advantages, such as complementary metal-oxide-semiconductor (CMOS) implementation, low manufacturing temperatures, and promise when used as *n*-type or *p*-type semiconductors [5].

Until now, most studies on oxide semiconductors have focused on *n*-type oxide semiconductors. A hydrogenated polycrystalline indium oxide TFT grown through a recently studied low-temperature solid-state hardening process has excellent switching characteristics with a field effect mobility of 139.2 cm^2^ V^−1^ s^−1^, a threshold swing of 0.19 Vdec^−1^, and a threshold voltage of 0.2 V [6]. Bae et al. also studied solution-processed indium–zinc oxide (IZO) TFTs on flexible polyethylene naphthalate substrates via fluorine doping at a low temperature (200 °C). The fluorine dopant increases the carrier concentration, allowing IZO TFTs with relatively high mobility (4.1 cm^2^ V^−1^ s^−1^) to be developed even at low temperatures [7].

The demand for *p*-type oxide semiconductors that are comparable to *n*-type oxide semiconductors is increasing for CMOS circuits [8]. Several studies have been conducted to demonstrate the working of *n*-type oxide semiconductor TFTs. However, it is difficult to fabricate a high-performance *p*-type oxide semiconductor because the valence band maximum (VBM) of the *p*-type oxide semiconductor is mainly composed of anisotropic and localized oxygen 2p orbitals, the hole effective mass is large, and the mobility is low [9,10,11]. Therefore, *p*-type oxide semiconductors are inferior in performance to *n*-type oxide semiconductors, preventing their wide application. The development of high-performance *p*-type oxide semiconductors is essential for realizing next-generation electronic products with excellent transparency and high power efficiency.

Several studies have been conducted to improve the electrical performance of *p*-type oxide semiconductors. Using gallium doping cuprous oxide (Cu_2_O) TFTs, Bae et al. improved the *p*-type electrical properties by reducing oxygen vacancies and hindering hole carrier transfer [12]. Kim et al. engineered a *p*-type tin oxyselenide (SnSeO) material by hybridizing Se 4p orbitals with SnO to further delocalize the VB edge, resulting in improved electrical performance [13]. Recently, Zeng et al. also investigated the performance of CuO TFTs, reporting an on/off current ratio of 1.3 × 10^2^ and a field effect mobility of 1.1 × 10^−3^ cm^2^ V^−1^ s^−1^ [14]. In order to demonstrate oxide-semiconductor-based CMOS devices, the electrical properties of *p*-type oxide semiconductors still need to be improved in comparison with those of *n*-type oxide semiconductors.

The post-processing technology used in this study has the advantage of not being limited by the material or composition, thus enabling further research. Seo et al. improved the mobility of zinc–tin oxide TFTs by removing impurity ions and creating oxygen vacancies through post-annealing under a vacuum and wet air [15]. Faber et al. enhanced the switching characteristics and field effect mobility (up to 23.8 cm^2^ V^−1^ s^−1^) in a TFT device by treating the activated zinc oxide layer with oxygen plasma, which eliminated defects and dissolved the acetate ligand shell of the particles [16]. Post-processing using vacuum and plasma is difficult and relatively expensive over a large area. Therefore, a simple method is required.

In this study, an active layer was fabricated from a copper oxide (CuO) thin film using a solution process. The solution process enables cost-effective manufacturing that minimizes material waste through area-selective deposition within a thin film. Furthermore, it is easy to fabricate oxide semiconductors using solution processes owing to advantages such as simplicity, easy composition control, and high throughput. However, it has several disadvantages, such as a high thermal budget and low stability, and research is being conducted to address these drawbacks.

The effect of short-term post-UV/O_3_ treatment on the crystal and electrical properties of solution-processed CuO thin films was investigated. The fabricated thin films were analyzed using thermogravimetric analysis (TGA), absorbance measurements, atomic force microscopy (AFM), X-ray photoelectron spectroscopy (XPS), Hall effect measurements, and Raman spectroscopy. In addition, the performance was studied by analyzing the output and transfer characteristics of CuO-based TFTs.

## 2. Materials and Methods

**Figure 1:**
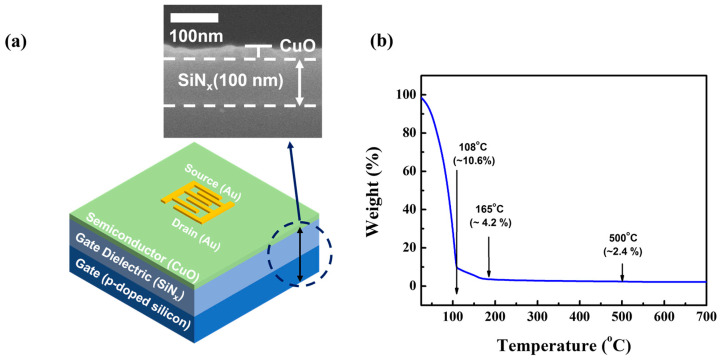
(**a**) Schematic representation of the fabricated CuO TFT. The inset represents the cross-sectional SEM image. (**b**) TGA characteristic curve of the prepared CuO precursor solution.

Figure 1 shows the structure of the fabricated solution-processed CuO TFT with a bottom-gate/top-contact structure (Figure 1a). The TFT was fabricated using a boron-doped *p*-type silicon wafer as a substrate, on which a 100-nm-thick silicon nitride (SiN_x_) dielectric layer was deposited; the electrical resistivity of the present *p*-type silicon wafer was as low as 1 to 10 Ωcm, so the substrate could be used as the gate electrode for the TFTs in this study. A precursor solution was created by mixing 0.4 M of copper (II) acetate hydrate [Cu(CO_2_CH_3_)_2_H_2_O] with 0.8 M of mono-ethanolamine [NH_2_CH_2_CH_2_OH] in 5 mL of 2-methoxyethanol [CH_3_OCH_2_CH_2_OH], which was then agitated for 1 h at 75 °C with a rotation speed of approximately 750 rpm. The prepared solution was subjected to air aging for 24 h. To increase the coatability of the solution on the substrate, an oxygen plasma treatment was used to make the substrate surface hydrophilic. A radio frequency power of 45 W was applied for 2 min during the oxygen plasma treatment, while the oxygen flow rate was held constant at 20 sccm. The solution was filtered through a poly-tetrafluoroethylene syringe filter with a pore size of 0.2 μm and spin-coated on the treated substrate at 2000 rpm for 35 s. After drying on a hotplate at 80 °C for 5 min and 150 °C for 20 min to evaporate the solvent, the coated film was thermally annealed at 500 °C for 30 min in a vacuum tube furnace. The solution-processed CuO semiconductor films were used for TFT fabrication without a patterning process. Then, a 30-nm-thick Au source and drain electrodes were thermally deposited over the CuO semiconductor layer through a shadow mask at a base pressure of approximately 6 × 10^−6^ Torr. The interdigitated electrodes were composed of five pairs, each with an electrode width of 400 μm and a channel length of 80 μm. Therefore, the effective channel length and width of the transistors were 80 and 2000 μm, respectively. It should be noted that the fringing field issue has become a problem when TFTs with bottom-gate/top-contact structures have un-patterned semiconductor layers as well as bar-type line S/D electrodes. In particular, TFTs with a bottom-gate/top-contact structure exhibit non-saturated drain currents in their output characteristics because fringe-field-induced currents are produced at the semiconductor surface between the source and drain electrodes; such currents at the semiconductor surface are not likely to be controlled by the gate voltage. The interdigitated structure of the source and drain electrodes is effective at avoiding (or minimizing) the fringing field issue in TFTs with a bottom-gate/top-contact structure; the effect of the fringing field can be reduced by expanding and overlapping the area of the effective channel. Finally, post-UV/O_3_ treatment was performed for 1–13 min.

TGA measurements were performed (N-1000, Sinco, Republic of Korea) and the TGA curve of the CuO precursor solution is shown in Figure 1b. The evaporation of the 2-methoxyethenol solvent caused the majority of the weight loss (>90%) of the precursor solution as the temperature rose to approximately 108 °C. The rate of weight loss significantly slowed as the temperature increased from 180 to 165 °C. This indicates that Cu(CO_2_CH_3_)_2_H_2_O was hydrolyzed to Cu(OH)_2_. The rate of weight loss decreased when the temperature increased beyond 165 °C, and it is thought that, at these temperatures, Cu(OH)_2_ dehydrated to create CuO. Particularly at temperatures greater than 500 °C, there was no appreciable change in weight. As a result, 500 °C was found to be the ideal annealing temperature for creating CuO semiconductor films.

The influence of post-UV/O_3_ on the morphological and structural characteristics of the fabricated CuO films was investigated using AFM (Multimode IVa, Bruker, Billerica, MA, USA). Crystallographic properties were characterized using Raman spectroscopy (LabRAM HR Evolution, Horibe Scientific, Kyoto, Japan). Furthermore, the atomic bonding characteristics of the prepared CuO thin films were analyzed using XPS (K-alpha, ThermoFisher, Seoul, Republic of Korea). Hall effect measurements (HMS 3000, Ecopia, Anyang, Republic of Korea) were performed using the van der Pauw method to measure the carrier concentration, Hall mobility, and conductivity of the solution-processed CuO thin films. Finally, a semiconductor analyzer (4200-SCS, Keithley, Seoul, Republic of Korea) was used to assess the electrical properties of the fabricated CuO TFTs.

## 3. Results and Discussion

### 3.1. Optical Characteristics

**Figure 2:**
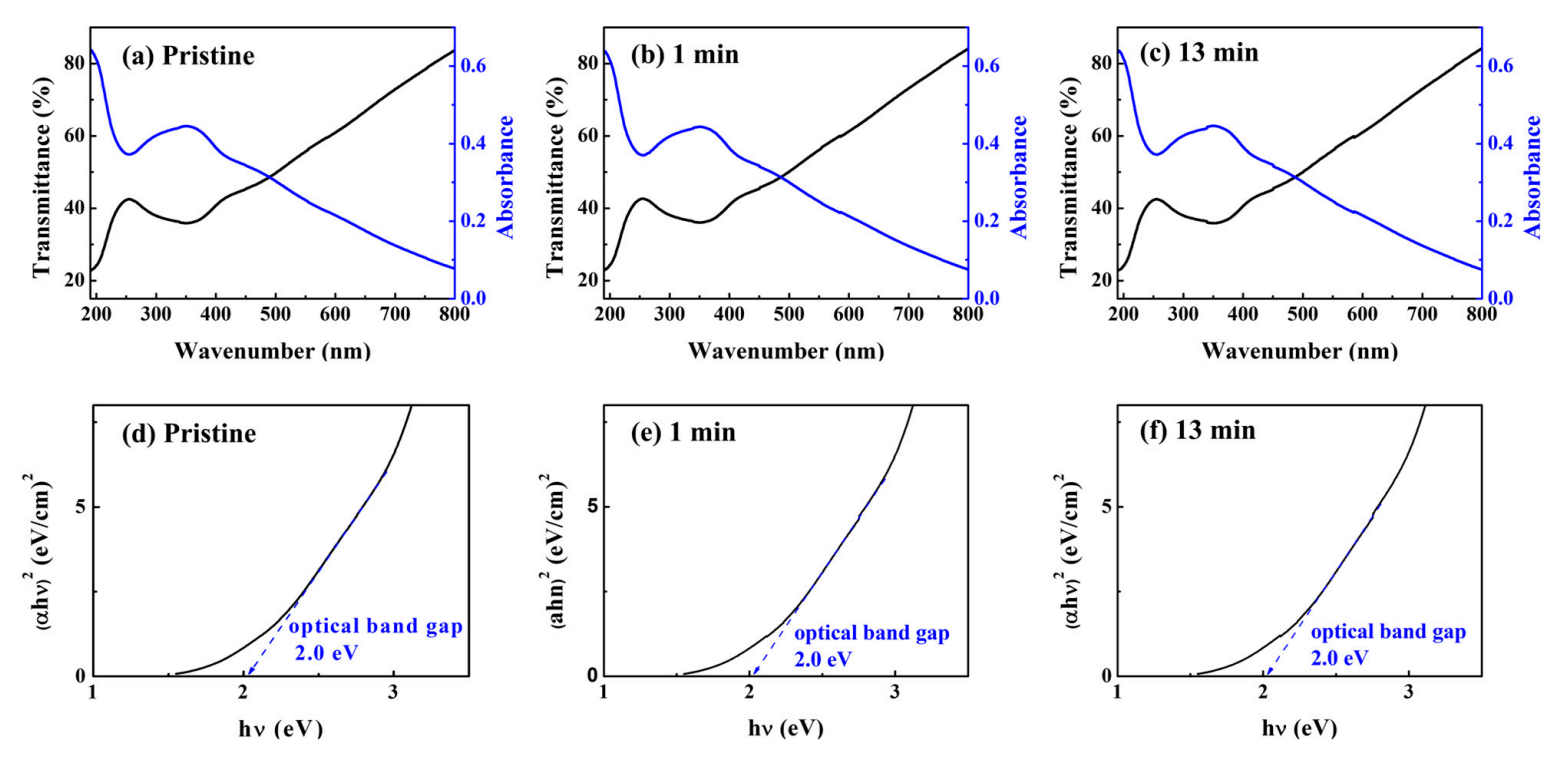
Optical transmittance and absorbance spectra of the solution-processed films with post-UV/O_3_ treatment for (**a**) pristine, (**b**) 1, and (**c**) 13 min. Determination of the optical band gap of the solution-processed films with post-UV/O_3_ treatment for (**d**) pristine, (**e**) 1, and (**f**) 13 min using Tauc plots.

The optical properties of the solution-processed CuO film analyzed using ultraviolet/visible (UV/vis) spectroscopy are shown in Figure 2. CuO films were coated on a quartz substrate and annealed at 500 °C in a vacuum environment for 30 min after soft baking on a hotplate for optical characterization. Figure 2a–c depict the transmittance of the CuO thin films in the 200–800 nm wavelength range. The transmittance of the films was ~80%. Figure 2d–f illustrate the optical band gap (E_g_) of the CuO films as calculated from the Tauc plot of the absorption graph ((αhν)^2^ plotted against hν), where hν is the input photon energy and α is the absorption coefficient. The absorption coefficient was determined by α = [2.303 × log(1/*T*)]/d, where *T* denotes the transmittance and d denotes the film thickness. Using Tauc’s relation, the E_g_ of the films is calculated, as (αhν)^2^ ∝ (hν—E_g_), where h is Planck’s constant and ν is the photon frequency [17]. The optical band gaps of the pristine CuO films and (1 and 13 min) UV/O_3_-treated CuO films were found to be 2.02 eV. According to the literature, the optical band gap of CuO is 1.3 eV–2.1 eV [18]. 

### 3.2. Structural and Stoichiometric Characteristics

**Figure 3:**
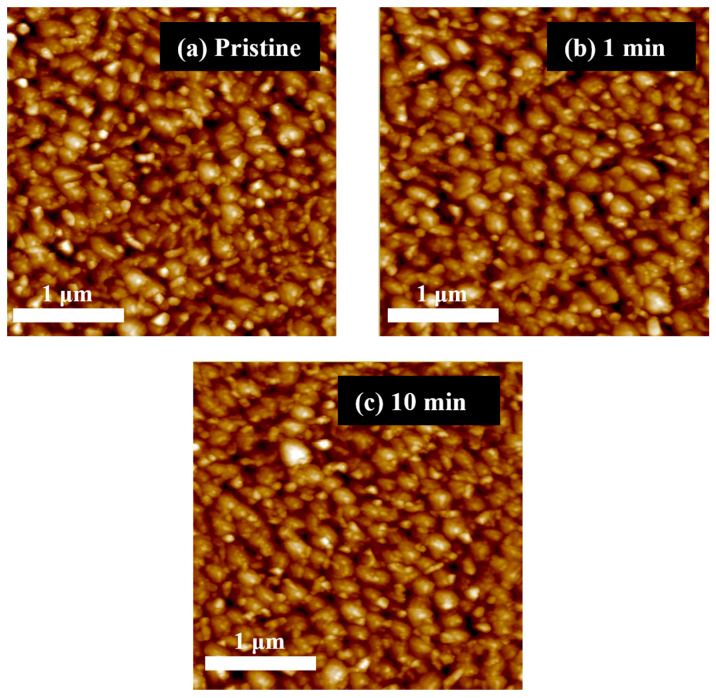
AFM images (1 μm × 1 μm) of solution-processed CuO thin films with post-UV/O_3_ treatment for (**a**) pristine, (**b**) 1, and (**c**) 13 min.

The surface morphologies of the pristine and post-UV/O_3_-treated CuO films were examined using AFM and are shown in Figure 3. The surface morphologies of all the films were comparable, indicating that the solution-processed CuO films were not significantly altered by the post-UV/O_3_ treatment in terms of pore formation, delamination, or cracking. However, the CuO film became slightly smoother as the post-UV/O_3_ treatment period increased. The CuO films were exposed to UV light for 0, 1, and 10 min, and the resulting root-mean-square roughness values were approximately 7.5, 7.4, and 6.9 nm, respectively. It was confirmed that no significant changes occurred on the film surface after UV/O_3_ treatment.

**Figure 4:**
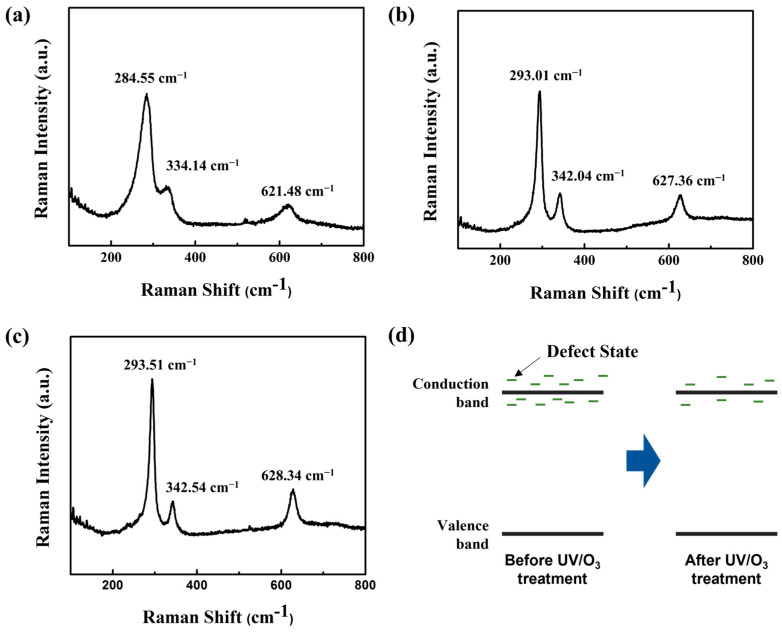
Raman spectra of solution-processed CuO films with UV/ozone treatment for (**a**) pristine, (**b**) 1, and (**c**) 13 min. (**d**) The energy band diagram and energy state distribution of CuO semiconductor before and after UV/O_3_ treatment.

By employing Raman spectroscopy, we further investigated how post-UV/O_3_ treatment affected the lattice structure of the solution-processed CuO films. For the experiment, the laser spot size was maintained at approximately 1 μm, and the wavelength of the excitation laser beam was held at 532 nm. The Raman spectra of the CuO films that underwent post-UV/O_3_ treatment for 0, 1, and 13 min are shown in Figure 4. Raman peaks were measured for the pristine CuO film at approximately 284.6 cm^−1^, 334.1 cm^−1^, and 621.5 cm^−1^. Raman peaks of the CuO film exposed to post-UV light for 1 min and 13 min were seen at approximately 293.0/342.0/627.3 cm^−1^ and 293.5/342.5/628.3 cm^−1^, respectively. These Raman characteristic peak wavenumbers were comparable to those reported in the literature. The peak at 284.6/293.0/293.5 cm^−1^ can be attributed to the A_g_ mode, whereas the peaks at 334.1/342.0/342.5 cm^−1^ and 621.5/627.4/628.3 cm^−1^ can be attributed to the B_g_ mode of CuO [19,20]. The Raman peak location changes toward a higher wavenumber as the fabricated CuO film is exposed to UV radiation, which is significant. Because compressive and tensile stresses can both account for moving to higher and lower wavenumbers, respectively, the shift in the peak position to a higher wavenumber suggests that the CuO film was compressed by UV irradiation [21,22]. It is assumed that this is because the post-UV/O_3_ treatment reduced the number of point defects (Figure 4d).

**Figure 5:**
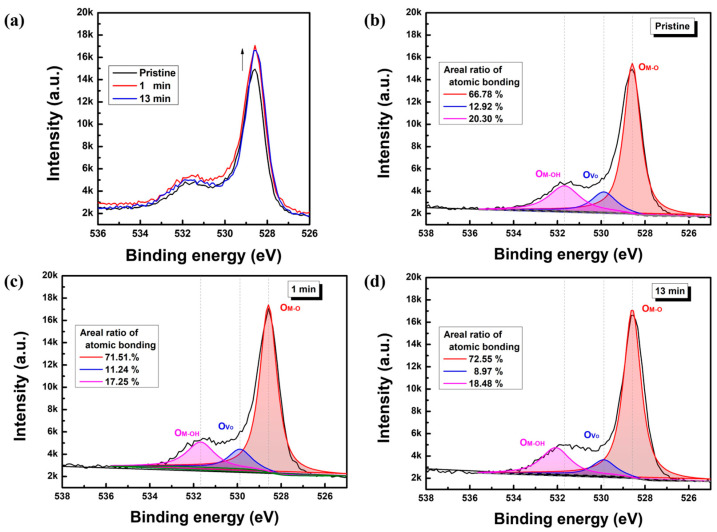
(**a**) The XPS spectra for analysis of binding energy and stoichiometric characteristics of solution-processed CuO semiconductor films. The deconvoluted O 1s spectrum of (**b**) pristine, (**c**) 1 min, (**d**) 13 min post-UV/O_3_-treated CuO film. The insets of (b-d) represent the atomic composition ratios of the bonds.

Based on the Raman spectrum results, the XPS measurement was performed to analyze the stoichiometric characteristics of solution-processed CuO semiconductor films, as shown in Figure 5. Figure 5a compares the XPS O 1s spectra of pristine and UV/O_3_-treated CuO, and Figure 5b–d analyze the O 1s main peaks of the pristine and UV/O_3_-treated films, respectively. The XPS characteristics of CuO films were measured after etching a 10 nm CuO film from the surface. The O 1s main peaks in Figure 5b–d were observed approximately at 528.58, 529.88, and 531.68 eV, respectively; these peaks represent the metal–oxygen (M–O) ionic bonding, oxygen vacancy (V_O_), and metal–hydroxyl (M–OH) impurity bonding energies [23,24,25], respectively. As shown in Figure 5b–d, the atomic composition ratio of the M–O bonding in Figure 5b was approximately 66.78%, which was 4–5% lower than those observed in Figure 5c, i.e., 71.51%, and Figure 5d, i.e., 72.55%. The composition ratio of V_O_ and M–OH bonds was not significantly changed. The XPS results highlight that the post-UV/O_3_ treatment intimately affected the metal–oxygen bonding nature. Similar to the Raman analysis showing that the CuO lattice structure could be compressed by post-UV/O_3_ treatment, we found that the atomic composition concentration of M–O lattice bonding in solution-processed CuO semiconductor films was meaningfully increased by post-UV/O_3_ treatment. Therefore, it is reasonable to speculate that point defects near the band gap edges (conduction and/or valance bands), which are known to arise due to weak bonding and structural defects between Cu and O bonds, are suppressed by post-UV/O_3_ treatment, thereby being replaced by M–O bonds [26,27]. Accordingly, it is found that the superimposed s-orbital of the metal atoms is further enhanced by the compressed atomic structure, and the electrical properties of solution-processed CuO semiconductors can be changed even with a small variation change of 4–5%.

### 3.3. Electrical Characteristics

Table 1 compares the hole–carrier concentration, Hall mobility, and conductivity of the CuO films that were subjected to the post-UV/O_3_ treatment for varying durations. The hole–carrier concentrations of the solution-processed CuO films before and after post-UV/O_3_ treatment were comparable at 1.05 × 10^15^ cm^−3^ and 1.10 × 10^15^ cm^−3^, respectively. The hole–carrier concentration was within the error range. On the other hand, the Hall mobility and conductivity significantly increased from 96.7 cm^2^ V^−1^ s^−1^ to 280 cm^2^ V^−1^ s^−1^ and from 4.35 × 10^−3^ Ω^−1^ cm^−1^ to 4.57 × 10^−2^ Ω^−1^ cm^−1^. These variations in hole–carrier concentration, Hall mobility, and conductivity confirm that the structural modification of solution-processed CuO films due to post-UV/O_3_ treatment can also enhance the electrical properties of the films [28].

**Figure 6:**
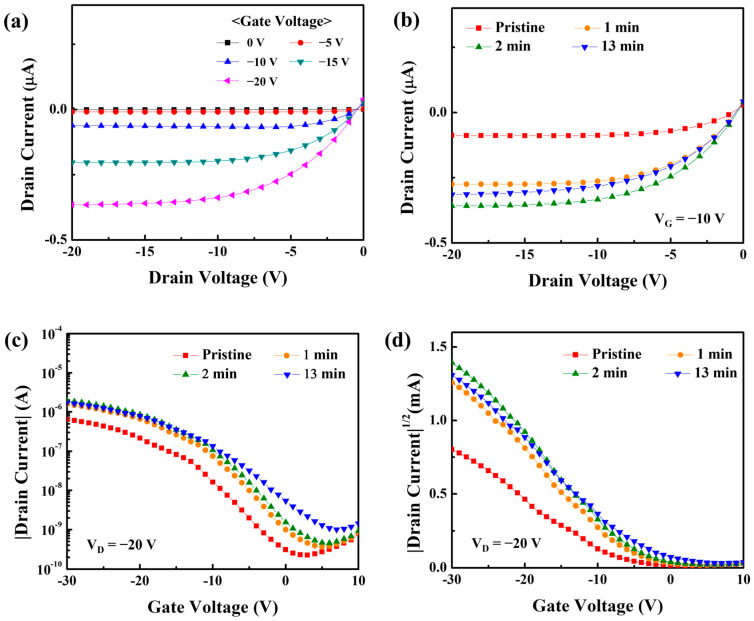
Output characteristics (I_D_ versus V_D_ plots) of (**a**) the pristine and (**b**) UV/O_3_-treated CuO TFTs. Transfer characteristics ((**c**) |I_D_| and (**d**) |I_D_|^1/2^ versus V_G_ plots) of the solution-processed CuO TFT with UV/O_3_ treatment for pristine, 1, 2, and 13 min.

To examine the impact of UV/O_3_ treatment on the performance of the solution-processed CuO TFT, the electrical characteristics of the transistor were examined as a function of the UV/O_3_ treatment period. Figure 6a shows the output characteristics of the post-UV/O_3_-treated CuO TFT when the drain voltage (V_D_) was changed from 0 V to −20 V in −1 V increments at a gate voltage (V_G_) from 0 V to −20 V in −5 V increments. Under p-channel accumulation-mode operation, the CuO TFT displayed a visible pinch-off behavior and thus good saturation in currents, demonstrating that holes predominated as charge carriers in the CuO semiconductor layer. It is observed that the interdigitated source/drain structure in the fabricated transistors is effective for the saturation behavior of top-contact/bottom-gate structured TFTs by reducing the fringing field effect at the semiconductor surface. Furthermore, Figure 6b reveals that post-UV/O_3_ treatment can improve the output qualities of solution-processed CuO TFTs. Although the drain current (*I_D_*) of the post-UV/O_3_-treated TFT was greater, the pinch-off and saturation behaviors could still be maintained without deterioration. *I_D_* (*V_D_* = −20 V, *V_G_* = −10 V) increased by nearly 3.1 times after receiving UV exposure for 1 min. When exposed to UV light for 2 min, *I_D_* (*V_D_* = −20 V, *V_G_* = −10 V) increased by 4.4 times. However, when the treatment period was increased by more than 10 min, the current did not increase notably. The result suggests that the post-UV/O_3_ treatment may be sufficient to improve the TFT performance even if it is performed for a short period of time, such as less than 10 min. The transfer properties of the solution-processed CuO TFT are shown in Figure 6c–d in relation to the post-UV/O_3_ treatment period; herein, the electrical characteristics of solution-processed CuO TFTs were evaluated using more than 20 transistors manufactured under each condition to analyze the effect of post-UV/O_3_ treatment. While the gate voltage was swept reversibly in steps of −1 V from 10 V to −30 V, these properties were measured at a fixed drain voltage of −20 V. The subthreshold (S.S.), which is defined as the change in gate voltage necessary to change the drain current by a factor of 10, was taken from the plot of |*I_D_*| versus *V_G_* (Figure 6c) to assess the performance of the TFT. The threshold voltage (*V_T_*) was obtained by extrapolating the plot of |*I_D_*|^1/2^ vs. *V_G_* to a drain current of 0 A (Figure 6d). The field effect mobility (*μ_eff_*) in the saturation region was calculated using the following equation:

$$I_{D}=\frac{W\mu_{eff}C_{dielectric}}{2_{L}}{\left(V_{G}-V_{T}\right)}^{2}$$

where *C_dielectric_* is the capacitance per unit area of the gate dielectric layer; the capacitance value of approximately 60.47 nFcm^−2^ was used in this study, which was estimated when the dielectric constant and thickness of the silicon nitride dielectric layer were 6.8 and 100 nm, respectively [29]. Table 2 summarizes the TFT parameters. The pristine CuO TFT (UV/O_3_ treatment period of 0 min) had a *V_T_* of −7.32 V, *μ_eff_* of 1.81 × 10^−3^ cm^2^V^−1^s^−1^, and on/off current ratio (I_ON/OFF_) of 2.83 × 10^3^. The *V_T_* of the UV/O_3_-treated for 1 min CuO TFT was −5.97 V, the *μ_eff_* was 4.38 × 10^−3^ cm^−2^V^−1^s^−1^, and the I_ON/OFF_ was 4.29 × 10^3^. The CuO TFT UV/O_3_-treated for 2 min exhibited a *V_T_* of −5.12 V, a *μ_eff_* of 4.97 × 10^−3^ cm^2^V^−1^s^−1^, and an I_ON/OFF_ of 4.26 × 10^3^. The TFT treated for more than 10 min exhibited a *V_T_* of −3.76 V, a *μ_eff_* of 3.82 × 10^−3^ cm^2^V^−1^s^−1^, and an I_ON/OFF_ of 1.69 × 10^3^. The *μ_eff_* of the UV/O_3_-treated CuO TFTs is rather impressive when compared to the 1.1 × 10^−3^ cm^2^V^−1^s^−1^ *μ_eff_* reported in the performance enhancement research for precursor-based solution-processed *p*-type CuO TFTs [14]. For a more accurate analysis of field effect mobility, it is necessary to prevent the formation of a depletion layer at the silicon nitride/silicon interface by using an *n*-type silicon substrate. It is also necessary to experimentally extract capacitance–voltage characteristics from devices fabricated by patterning the solution-processed CuO semiconductor layers.

According to the Hall effect results in Figure 6, UV/O_3_ treatment improved the electrical characteristics of the CuO semiconductor layer, which could explain the decrease in threshold voltage and increase in field effect mobility. As confirmed from the structural and stoichiometric properties of the Raman spectrum, XPS, and AFM analysis, the effects of the post-UV/O_3_ treatment on the electrical behavior of the solution-processed CuO TFT can be summarized as follows. Through the post-UV/O_3_ treatment, the atomic structure of the CuO semiconductor can be slightly compressed and the atomic composition ratio of the M–O bonding can be enhanced by 4–5% without critical damage to the film. Furthermore, the electrical enhancement of the Hall mobility can be attributed to the reduction in the point defects and the increase in the s-orbital overlap of metal atoms. Consequently, the electrical performance of *p*-type CuO TFTs can be improved by post-UV/O_3_ treatment due to the reinforced atomic structure and enhanced electrical conductivity. It is expected that post-UV/O_3_ treatment will be a novel technology to reduce structural defects in solution-processed *p*-type oxide semiconductors.

## 4. Conclusions

In summary, the effects of post-UV/O_3_ treatment on the structural and electrical properties of *p*-type CuO semiconductors and the performance of CuO TFTs were investigated. Through the structural and stoichiometric characterization of solution-processed *p*-type CuO films, it was determined that the post-UV/O_3_ treatment reduces weak bonds and structural defects in the M–O bonds and compresses the s-orbital overlap of the metal atoms. Owing to the post-UV/O_3_ treatment, the Hall mobility and conductivity significantly increased compared to the increased rate of the hole–carrier concentration of the CuO thin film. The electrical properties of solution-processed CuO TFTs were improved through post-UV/O_3_ treatment; the output current and field effect mobility were improved by a factor of approximately two through the post-UV/O_3_ treatment. This was explained by the reduction of point defects. It is concluded that the post-UV/O_3_ treatment can stoichiometrically change the M–O bonding nature of the solution-processed *p*-type oxide semiconductors, and, thus, it plays an important role in improving the electrical performance of the CuO TFTs. We believe that the post-UV/O_3_ treatment can be used to improve the performance of *p*-type oxide TFTs, regardless of material compositions as well as process conditions. To demonstrate oxide-semiconductor-based CMOS circuits, further studies are necessary to control the subthreshold behaviors of *p*-type oxide TFTs, which can be achieved by modifying the insulator–semiconductor interface characteristics and adjusting the difference in the metal–semiconductor work function.

## Figures and Tables

**Table 1:** Electrical properties of pristine and post-UV/O_3_-treated CuO semiconductors by Hall effect measurements.

	Bulk Concentration (10^15^ cm^−3^)	Hall Mobility (cm^2^ V^−1^ s^−1^)	Conductivity (10^−3^ Ω^−1^ cm^−1^)
Pristine	1.05 ± 0.7	96.7 ± 39.8	4.4 ± 2.9
Post-UV/O_3_ treatment	1.10 ± 0.2	280 ± 99.3	45.7 ± 19.7

**Table 2:** Summary of performance parameters for the pristine and UV/O_3_-treated CuO TFTs.

UV/Ozone Treatment Period (min)	*V_T_* (V)	I_ON/OFF_	*μ_eff_* (cm^2^ V^−1^ s^−1^)
Pristine	−7.32	2.83 × 10^3^	1.81 × 10^−3^
1	−5.97	4.29 × 10^3^	4.38 × 10^−3^
2	−5.12	4.26 × 10^3^	4.97 × 10^−3^
13	−3.76	1.69 × 10^3^	3.82 × 10^−3^

## Data Availability

The data presented in this study are available on request from the corresponding author.
